# FinFakeBERT: financial fake news detection

**DOI:** 10.3389/frai.2025.1604272

**Published:** 2025-12-03

**Authors:** Bledar Fazlija, Ismet Bakiji, Visar Dauti

**Affiliations:** 1 School of Management and Law, Zurich University of Applied Sciences, Winterthur, Switzerland; 2 Independent Researcher, Zurich, Switzerland

**Keywords:** fake news detection, financial fake news, domain shift, large language models, machine learning

## Abstract

The intentional use of fake news for financial manipulation or the disruption of financial markets is a serious concern, particularly with the rise of generative artificial intelligence, which is expected to significantly increase its dissemination. A lack of open-access, labeled *financial* fake news data poses challenges when training effective models for financial fake news detection. To address these challenges, we present FinFakeBERT, a family of models trained using newly curated fake news data. We demonstrate that fine-tuning BERT with a small set of actual fake financial news, following fine-tuning with a large cross-domain fake news dataset and accurate financial news articles, leads to high fake news detection accuracy and significantly reduces the false positive rate (FPR) when tested on several large sets of real financial news articles. Our best model achieves a 2.1% false positive rate (FPR) on real financial news, whereas available benchmark fake-news detectors exhibit FPRs that are more than three to ten times higher.

## Highlights

- We present FinFakeBERT, a financial fake news detection model based on BERT, fine-tuned using both cross-domain fake news data and domain-specific financial news.
- We investigated the impact of domain shift—the performance drop when a model trained with general fake news is applied to financial fake news.
- We collected verified financial fake news data using information from SEC indictments.
- BERT, fine-tuned with financial fake news data, significantly reduces the false positive rate (FPR) when predicting real financial news.

## 1 Introduction

The simplicity with which information is disseminated online has catalyzed the spread of fake news^1^ (i.e., fabricated or inaccurate news). In recent years, fake news has fueled political crises, heated societal debates, and ignited dangerous or even deadly riots. Fake news is also used to manipulate or disrupt financial markets. In this paper, we investigate the detection of financial fake news using language models fine-tuned with cross-domain and domain-specific data.

Fake news requires special attention as it spreads significantly further, deeper, faster, and more comprehensively than accurate information (Vosoughi et al., 2018). Indeed, there is evidence that this is also the case for financial fake news. According to Clarke et al. (2021), financial fake news attracts significantly more attention from investors than accurate news, with an average of 83.4 percent more page views. However, they found that article commentators are generally unable to identify fake news. Similarly, Kogan et al. (2023) observed that fake news increased retail investor trading volume by more than 55 percent in the three days following publication, compared to accurate articles by the same author. They further showed that exposure of fraud on social financial news sites, as revealed through a U.S. Securities and Exchange Commission (SEC) investigation led to a significant decrease in trading activity due to a loss of investor trust, even when the news was accurate.

Misinformation impacts stock prices and undermines investor confidence, resulting in a $130 billion loss in stock value within minutes of a false report claiming that Barack Obama had been injured in an explosion (Rapoza, 2017). More recently, in April 2025, fake news about a supposed delay in tariffs by the U.S. Administration caused the S&P 500 to surge by 8.5% in about half an hour, adding $3.6 trillion in market value.^2^

Given the clear and often detrimental implications of fake news, the automated detection of financial fake news has become critically important. Recent advances in the development of large language models (LLMs) are likely to accelerate both the dissemination of fake news (Vinay et al., 2025) and enhance efforts to detect it, owing to their impressive capabilities in text generation and understanding, as well as their accessibility, which requires little technical expertise. As noted by Spitale et al. (2023), GPT-3 can generate fake news (in the form of tweets) more convincing than that written by humans, making it impossible to distinguish reliably between the two.

There are some studies on the detection of financial fake news, and Appendix Table B10 summarizes some of them. They present three main lines of research for (financial) fake news detection.

The first uses “classical” models, often based on linguistic features or semantic and syntactic analyses (Chung et al., 2023; Zhang et al., 2022; Clarke et al., 2021; Kogan et al., 2023). For more general discussions about such methods, refer to the following surveys (Chen et al., 2015; Shu et al., 2017; Zhou and Zafarani, 2020; De Beer and Matthee, 2021).

The second involves training machine learning models using data collected from social media, online news outlets, or fact-checking websites (Mohankumar et al., 2023; Zhang and Liu, 2023; Zhi et al., 2021; Clarke et al., 2021; Zhang X. et al., 2020; Pérez-Rosas et al., 2017).

The third approach involves utilizing large pre-trained models for detecting financial fake news. This is done either through fine-tuning for the downstream task of fake news detection (Kamal et al., 2023; Zhi et al., 2021; Khan et al., 2021; Kaliyar et al., 2021; Sun et al., 2019) or deploying LLMs via prompting using the very large, recently developed LLMs (e.g., GPT-4). Quelle and Bovet (2024) use LLMs (GPT-3.5 and GPT-4) for fact-checking claims, showing good performance, with GPT-4 outperforming GPT-3.5.

These models usually have hundreds of millions or even billions of parameters and are adapted by fine-tuning, namely, adjusting (a subset of) their parameters based on a task-specific dataset (Murel and Kavlakoglu, 2024; Sun et al., 2019), or, for very large language models, issuing instructions by means of prompts to solve a task such as fake news detection.

Despite the clear relevance and growing interest in fake news detection, there are relatively few studies specifically on *financial* fake news and its impact on financial markets. We address this gap by proposing a domain-adapted transformer model (FinFakeBERT), a language model pre-trained using a large, unlabeled dataset (Devlin et al., 2019; Khurana et al., 2023). BERT comes in two variants: BERT_base, with 110 million parameters, and BERT_large, with 340 million parameters. BERT achieves high performance across various tasks by simply fine-tuning using a small amount of labeled data from the target domain (Ramponi and Plank, 2020), including natural language inference, question answering, sentiment analysis, topic modeling, and named entity recognition. Our resulting models, named FinFakeBERT, are not to be confused with FakeBERT (Kaliyar et al., 2021), which differs fundamentally in terms of model architecture, data used, and domain of application. Ours deals with financial fake news, while theirs concerns fake news on social media.

The success of ML-based fake news detection depends on the choice of model architecture and, crucially, on the quality and size of the training data (Sarker, 2021). ML models perform particularly well within the distribution of the training data, while generalization to new, unseen data is the ultimate goal. Poor results, according to Farahani et al. (2021), can occur when collecting training and test data from different sources or using a training dataset that has become outdated over time owing to changes in the data (Agarwal and Nenkova, 2022). For example, text data may differ in writing style and vocabulary between the training and test data (Elsahar and Gallé, 2019).

This discrepancy between the distributions of data in the source and target domains—known as “domain shift” can lead to worse prediction results when applying the trained model to a new dataset (Farahani et al., 2021; Quiñonero-Candela et al., 2022). In natural language processing (NLP), the term “domain” refers to a dataset characterized by specific features, such as topic, style, genre, or linguistic register (Ramponi and Plank, 2020). Calderon et al. (2024) examined the robustness of NLP models to domain shifts and showed that fine-tuned models suffer performance losses when switching domains.

One possible approach to investigating domain shifts is to monitor defined performance metrics [e.g., the false positive rate (FPR)] of the trained model in both the source and target domains (Calderon et al., 2024; Huang, 2023). Although the impact of domain shift has not been studied for financial fake news, there are studies on domain shifts in fake news detection, mostly focusing on domain adaptation before prediction, such as Zhang T. et al. (2020), or using multi-domain datasets and models designed for multi-domain prediction, such as Nan et al. (2021, 2022).

### 1.1 Research objectives

This study is motivated by two research questions:

- How effectively does BERT, when fine-tuned using cross-domain fake news data, perform in detecting financial fake news?
- How effectively can the impact of domain shift be mitigated in financial fake news detection, using a small set of actual financial fake news articles?

To this end, we constructed a large cross-domain fake news dataset, *Datacorpus* (*n* = 239,389), by aggregating several publicly available datasets. Using this to fine-tune BERT results in the model CDFakeBERT.

To address domain shift, we collected a new dataset of verified financial fake news [based on U.S. Securities and Exchange Commission (SEC) indictments] and supplemented it with accurately labeled financial news. We then fine-tuned CDFakeBERT using this domain-specific financial data and evaluated the impact of adaptation by computing the false positive rate of the resulting model, *FinFakeBERT*, on large sets of real financial news.

The remainder of the paper is structured as follows: Section 2 describes the methodology, including the datasets used and the mining methods employed, as well as the models trained on the different setups. Section 3 presents the accuracies and false positive rates of the models. Section 4 discusses the implications of the findings, limitations, and potential directions for future research. Appendix A provides a table with relevant studies on financial fake news detection.

## 2 Methodology

We conducted two phases of experiments. In Phase 1, we compared baseline models trained on the large dataset Datacorpus (cf. Section 2.1.1) to BERT-base-uncased fine-tuned on Datacorpus, leading to the model CDFakeBERT, where **CD** stands for **c**ross-**d**omain.

Although the optimal hyperparameters may vary depending on the task, the study by Devlin et al. (2019), which introduced BERT, showed that the following ranges are effective for various tasks: a learning rate between 2 × 10^−5^ and 5 × 10^−5^ with the Adam optimizer, a batch size of 16 or 32, and a training duration of 2 to 4 epochs. The researchers also found that large datasets with over 100,000 labeled data points were less sensitive to the choice of hyperparameters than small datasets.

In line with this, we fine-tuned all weights using the following hyperparameters: a batch size of 16, a learning rate of 1 × 10^−5^, and 5 epochs, selected through early stopping with a patience parameter set to 3. Training required 11 hours and 40 min on a DGX station equipped with four Tesla V100-DGXS-32GB GPUs.

**Figure 1:**
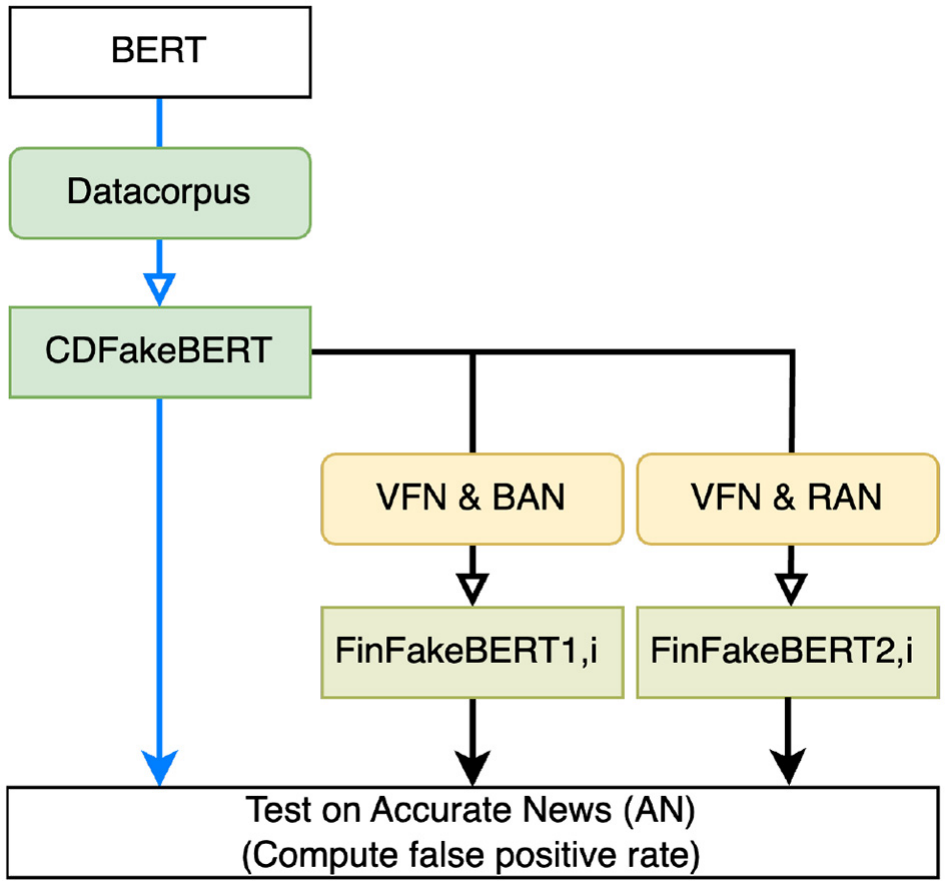
Phase 2: Fine-tuning different versions of BERT. BERT-base-uncased was fine-tuned using DataCorpus, resulting in CDFakeBERT, and tested on the data AN. CDFakeBERT was further fine-tuned using two separate sets of data, namely VFN (cf. Section 2.1.2) and BAN (cf. Section 2.1.4), and VFN and RAN (cf. Section 2.1.5), with different features (title, title+text, text), resulting in the sets of models FinFakeBERT1,i and FinFakeBERT2,i. All these models were tested using the FRP on AN.

Figure 1 provides an overview of Phase 2, during which we further fine-tuned CDFakeBERT using several data configurations, as described in the following sections. For Phase 2, the following parameters were used across the different fine-tuning scenarios: batch size of 4, learning rate of 1 × 10^−6^, number of epochs of 10, resulting in a runtime of 5–6 min for 10 epochs across the experiments due to the much smaller datasets used. In this phase, we used a smaller learning rate to avoid excessive bias toward the new, considerably smaller datasets while still capturing the nuances specific to financial fake news.

### 2.1 Datasets

#### 2.1.1 Datacorpus

Datacorpus is a unique cross-domain dataset of labeled, accurate, and fake news texts from various sources. Table 1 lists the various datasets and their characteristics.

**Table 1:** Overview of datasets used, including domain coverage and distribution of fake and accurate news entries.

**Data set**	**References**	**Domains**	**Fake**	**Accurate**	**Total entries**
1: Fake news	Ahmad et al., 2020	Politics, sports, entertainment	2,120	1,868	3,988
2: ISOT data set	Mishra et al., 2022; Samadi et al., 2021	Economy, politics, entertainment	23,481	21,417	44,898
3: LIAR data set	D'ulizia et al., 2021; Khan et al., 2021; Samadi et al., 2021; Wang, 2017	Economy, politics, healthcare	2,833	3,638	6,471
4: GM data set	Khan et al., 2021; Sastrawan et al., 2022	Economy, politics	3,164	3,171	6,335
5: Fake or real	Ahmad et al., 2020; Mishra et al., 2022	Economy, politics, entertainment	10,387	10,413	20,800
6: FN-set	-	Economy, politics	44,459	96,024	140,483
7: Guardian data set	-	Economy, politics, sports, art, technology, culture	52,462	0	52,462
8: Fakenews net	Harvard, 2022; Shu et al., 2020	Politics, social media	5,755	17,441	23,196
Total	-	-	144,661	153,972	298,633
After preprocessing	-	-	114,526	124,863	239,389

The LIAR dataset contains **10,239** articles, with six possible labels: “*true,” “mostly true,” “half true,” “barely true,” “false,”* and “*pants on fire.”* Only the articles labeled “*mostly true,” “true,” “false,”* and “*pants on fire”* were used.

In the preprocessing step, articles with fewer than 30 words and non-English articles were removed to reflect the goal of predicting whether English-language news articles are fake. In alignment with standard NLP preprocessing procedures, we further removed stop words (after extending the list with the most frequent words in the given texts), duplicates, HTML tags, emoticons, and punctuation. This resulted in a total of 239,389 articles, consisting of 124,863 accurate news items and 114,526 fake news items.

#### 2.1.2 Verified fake news

To identify verified financial fake news (VFN), indictments on the SEC website^3^ were reviewed, specifically those related to fraud, manipulation, and false press releases. News articles from the indicted companies were retrieved for the periods mentioned in the indictments. These articles were sourced from the companies' own websites and news platforms such as *Business Wire*,^4^ *PR Newswire*,^5^ *GlobeNewswire*,^6^ *AccessWire*,^7^ *PRLog*,^8^ *and Yahoo Finance*.^9^ For deleted articles, the Web Archive^10^ was used for retrieval. This process resulted in **233 fake news articles** from 16 different companies, published between 2009 and 2023.

#### 2.1.3 Accurate news

AN consists of the following three datasets. Table 2 highlights the used features.

**Figure 2:**
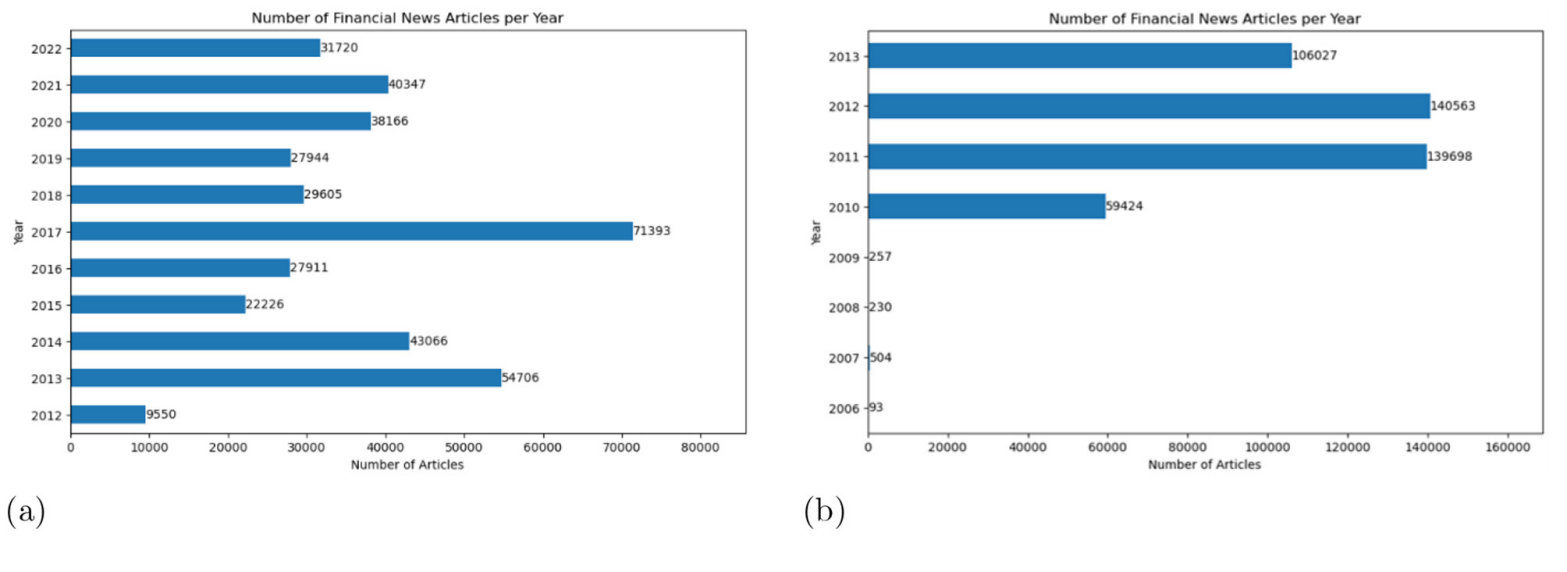
Temporal distributions of articles in the datasets **(a)** Motley Fool and **(b)** Bloomberg.

- **Bloomberg dataset (Philippe Remy**, **2015****):** This dataset contains 446,796 accurately labeled financial news articles from the financial news site *Bloomberg*,^11^ covering the period from 2006 to 2013. Figure 2 depicts the distribution of articles over time, showing that most articles in the dataset were from 2012 and 2011, followed by 2013.
- **Motley fool dataset:** This dataset, like the data corpus, was also provided by Dauti (2022). It includes 396,642 financial news articles from the financial news site *The Motley Fool*^12^ in a social media format, covering the period from 2012 to 2022. Figure 2 depicts the temporal distribution of articles in both the Motley and Bloomberg datasets.
- **Reuters dataset:** This dataset was also employed to examine domain shift after fine-tuning the BERT models across all phases. The Reuters dataset differs from the other datasets in that it exclusively comprises 8,556,310 accurately labeled financial news titles published on the financial news site *Reuters* between 2007 and 2016. For predictions on this dataset, entries with fewer than five characters—at total of 219 entries—were removed. No further preprocessing was conducted in this study.

**Table 2:** Dataset feature used for testing.

**Datasets**	**Title**	**Title + Text**	**Text**
Bloomberg	✓	✓	✓
Motley	✓	✓	–
Reuters	✓	–	–

#### 2.1.4 Bloomberg accurate news

BAN is a subset of *n* = 233 datapoints of the Bloomberg data contained in AN.

#### 2.1.5 Recent accurate news

**Figure 3:**
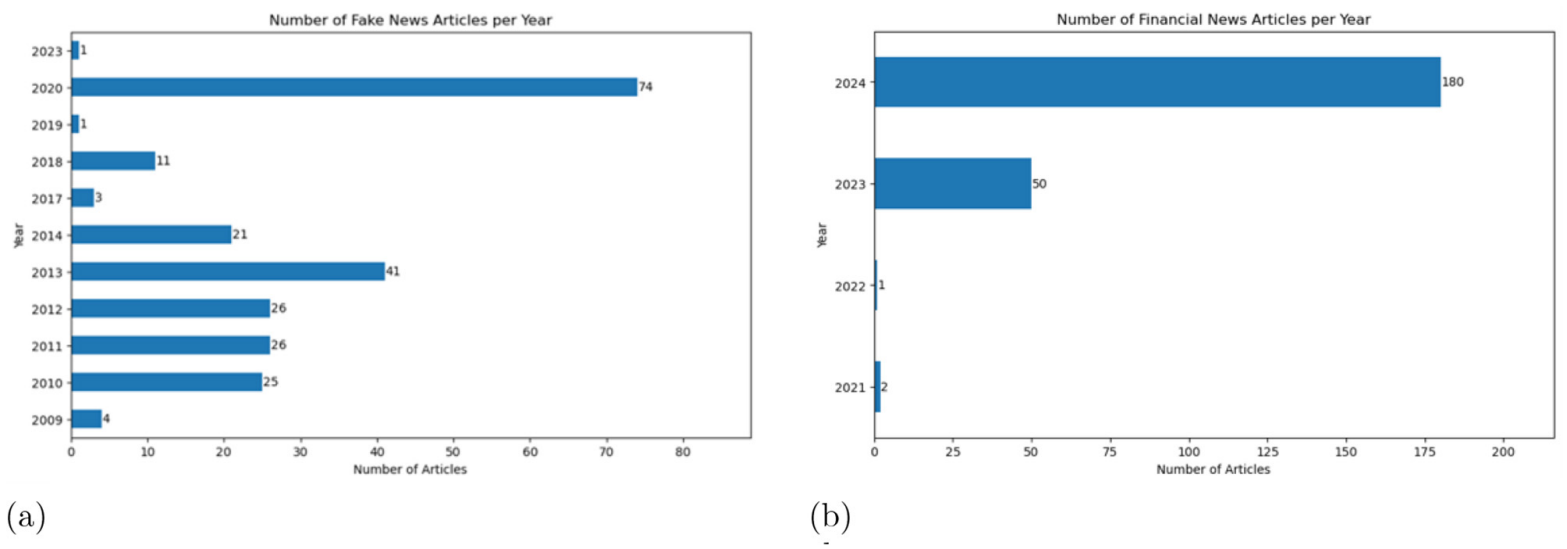
Temporal distributions of financial news articles in datasets **(a)** VFN and **(b)** RAN. **(a)** Temporal distribution of financial fake news articles in VFN. **(b)** Temporal distribution of real financial news articles in RAN.

RAN consists of 233 current and accurate financial news articles that were randomly selected from Bloomberg, Reuters, and *the Financial Times*.^13^ To create a balanced dataset, approximately equal numbers of articles were chosen from each platform: 80 from Bloomberg, 79 from Reuters, and 74 from the Financial Times, all published between 2021 and 2024. Figure 3 illustrates the distribution of fake and accurate news articles over time.

#### 2.1.6 Final pre-processing steps

For both fake and accurate texts, metadata such as contact information and the name of the news platform were removed. Since the texts varied in terms of special characters and spacing, consecutive dashes and excessive spaces were eliminated, ensuring a uniform and consistent format. The final number of datapoints for each dataset is reported in Table 3.

**Table 3:** Number of datapoints per dataset.

	**Datacorpus**		**Bloomberg**	**Motley fool**	**Reuters**	**Fake news dataset**	
	**Real**	**Fake**				**Real**	**Fake**
Count	124,863	114,526	446,796	399,622	8,556,325	233	233

Next, we examine the properties of the data used in detail.

### 2.2 Modeling

We trained several machine learning models in the following two phases.

#### 2.2.1 Phase 1

- We fixed a train-test-split of 90%/10% on Datacorpus (sufficient owing to the large amount of available data).
- We trained baseline machine learning models—a support vector machine (SVM), a deep neural network (DNN)—feed-forward neural network with two ReLU-activated hidden layers (512 and 128 neurons), a multinomial naive Bayes (MNB) classifier, a passive-aggressive classifier (PA), and a random forest classifier (RFC)—on the Datacorpus.
- We performed cross-domain fine-tuning of *bert-base-uncased* using the training dataset split from **Datacorpus**, resulting in the model **CDFakeBERT**.
- Accuracy, precision, and recall were computed on the test dataset.

#### 2.2.2 Phase 2

- We conducted domain-specific fine-tuning of **CDFakeBERT** on **VFN** and **BAN**. Models were fine-tuned with titles, texts, and titles + texts separately, resulting in the three models **FinFakeBERT1,i**, where *i* ∈ {title, text, title+text}.
- We conducted domain-specific fine-tuning of **CDFakeBERT** on **VFN** and **RAN** on titles, texts, and titles + texts separately, resulting in the three models **FinFakeBERT2,i**, where *i* ∈ {title, text, title+text}.
- Predictions were made on accurate news (AN), and the false positive rate (FPR)—i.e., the rate of accurate news falsely predicted as fake—is reported.

## 3 Results

We now compare the accuracy of different ML models on the available labeled data, “Datacorpus.” Given that news articles from reliable sources are considered legitimate and do not contain any fake news, we compute the false positive rate (FPR) for these articles and compare this measure across the different model variants that we fine-tuned using the data described above.

### 3.1 Results of Phase 1

The baseline models were trained on Datacorpus after a 90/10 train-test split. Similarly, bert-base-uncased was fine-tuned with the training part of Datacorpus, resulting in the model “CDFakeBERT.” All these models were evaluated on Datacorpus. Table 4 shows that BERT fine-tuned on Datacorpus achieved the best performance, with an accuracy of 98.6 percent, a precision of 98.9 percent, and a recall of 98.1%. The experiment with a 80/20 train-test split yields only marginally different results (Appendix Table 9).

**Table 4:** Comparison of baseline models [support vector machine (SVM), deep neural network (DNN), multinomial naive Bayes classifier (MNB), passive aggressive classifier (PA), and random forest classifier (RFC)] with the fine-tuned BERT model, using a 90/10 train–test split.

**Model**	**Accuracy**	**Precision**	**Recall**
CDFakeBERT	**98.6%**	**98.9%**	**98.1%**
SVM	96.0%	95.5%	95.3%
DNN	95.6%	94.7%	94.6%
PA	94.9%	94.7%	94.6%
MNB	88.7%	91.4%	84.3%
RFC	87.7%	90.0%	83.5%

Best results are highlighted in bold.

Additionally, CDFakeBERT was tested on the accurate news data AN, and the false positive rates for the different datasets that compose AN are presented in Table 5.

**Table 5:** False positive rate (FPR) across various datasets and configurations.

**Model**	**Dataset**	**Title**	**Title+Text**	**Text**
CDFakeBERT	Bloomberg	28.4%	3.6%	3.9%
	Motley	51.7%	4.8%	-
	Reuters	34.7%	-	-

CDFakeBERT (BERT fine-tuned in Phase 1 using the cross-domain data Datacorpus) was tested on AN.

### 3.2 Results of Phase 2

As mentioned in Section 2.2, CDFakeBERT was further fine-tuned with real and fake financial news, resulting in additional FinFakeBERT models. Table 6 presents the false positive rates of the different models evaluated on AN. The green cells in the table highlight cases where the FPR dropped relative to the results of CDFakeBERT presented in Table 5. Red cells depict cases with higher FPR.

**Table 6:** False positive rates across different datasets and fine-tuning configurations.

**Fine-tuning data**	**Model**	**Dataset**	**Title**	**Title+Text**	**Text**
VFN and BAN	FinFakeBERT1, title	Bloomberg	17.7%	3.0%	3.3%
		Motley	35.2%	5.4%	–
		Reuters	34.6%	–	–
	FinFakeBERT1, title+text	Bloomberg	23.2%	2.2%	2.5%
		Motley	49.5%	7.3%	–
		Reuters	38.2%	–	–
	FinFakeBERT1, text	Bloomberg	21.7%	2.0%	2.1%
		Motley	48.7%	8.0%	–
		Reuters	36.8%	–	–
VFN and RAN	FinFakeBERT2, title	Bloomberg	15.8%	3.1%	3.5%
		Motley	32.3%	3.8%	–
		Reuters	32.1%	–	–
	FinFakeBERT2, title+text	Bloomberg	19.7%	2.9%	3.2%
		Motley	43.0%	2.5%	–
		Reuters	34.6%	–	–
	FinFakeBERT2, text	Bloomberg	20.8%	4.2%	4.2%
		Motley	44.9%	3.1%	–
		Reuters	35.7%	–	–

Entries with a green background are cases where further fine-tuning of CDFakeBERT using the above-mentioned fine-tuning data reduced the FPR.

### 3.3 Comparison with benchmarks

To further evidence the potential utility of our approach, we conducted two additional experiments. In the first experiment, we applied *FinFakeBERT2, title+text*, which showed a reduction in false positive rates across datasets, to other publicly available benchmark datasets (WELFake and Fin-Fact, see Appendix B) and compared its performance to that of other models. The results are presented in Table 7. For this purpose, we used the following datasets.

**Table 7:** Performance comparison using WELFake and Fin-Fact datasets across different models.

**Source**	**Dataset, model**	**Accuracy**	**Recall**	**Precision**	**F1-score**
Our study	WELFake, FinFakeBERT2, title+text	91.3%	91.0%	91.7%	91.4%
Mohankumar et al. (2023)	WELFake, proposed model	–	94%	95%	94%
Our study	Fin-Fact, FinFakeBERT2, title+text	70.6%	89.8%	62.7%	73.9%
Rangapur et al. (2025)	Fin-Fact, GPT-4	78%	79%	76%	76%
	Fin-Fact, Claude3-Opus	64%	65%	62%	61%
	Fin-Fact, Gemini-Pro	47%	42%	45%	44%
	Fin-Fact, FMDLlama3	73.6%	73.6%	72.1%	73.6%

We now observe that our model performs well on new, unseen data and is typically only outperformed by some models proposed in the literature that were trained on the same dataset.

In the second experiment, we used the following openly accessible models for fake news detection from HuggingFace–selected based on relevance and the number of downloads.

- **Fake-News-Bert-Detect** ^14^
  This model is based on roberta-base (Conneau et al., 2019) and was fine-tuned using over 40,000 news articles from various media sources.
- **Bert-tiny-finetuned-fake-news-detection** ^15^
  This model is a fine-tuned version for fake news detection of bert-tiny (Turc et al., 2019; Bhargava et al., 2021).
- **Albert-base-v2-fakenews-discriminator** ^16^
  This model is a fine-tuned version of albert-base-v2 (Lan et al., 2019) using the dataset fake-and-real-news-dataset.^17^
- **Fake News Classification Distilbert** ^18^
  This model is based on the distilbert (Sanh et al., 2019), which was fine-tuned using the dataset fake-and-real-news-dataset (see text footnote ^17^).

We computed the false positive rates of these models on the Bloomberg dataset (see Section 2.1.3). The results are presented in Table 8.

**Table 8:** False positive rates (FPR) of fake news detection models.

**Model**	**FPR**
FinFakeBERT1, text	**2.1%**
FinFakeBERT1, title+text	2.5%
FinFakeBERT2, title+text	3.2%
FinFakeBERT2, text	3.5%
CDFakeBERT	3.9%
Albert-base-v2-fakenews-discriminator	7.6%
Bert-tiny-finetuned-fake-news-detection	11.0%
Fake-News-Bert-Detect	16.5%
Fake news classification distilbert	23.3%

Best results are highlighted in bold.

As shown in Table 8, our models exhibit a significantly lower false positive rate. This is crucial, as most published financial news is real news, making a low false positive rate particularly important for fake news detection models.

## 4 Discussion

Comparing our financial fake news detection models with those used in prior studies is a challenging task. This is due to differences in, and a lack of access to, the datasets used for training and evaluation, as well as the fact that most models are not easily accessible, for example, over Hugging Face.

Our study aims to contribute to the literature on fake news detection by focusing specifically on financial fake news. However, this presents two major challenges. First, there is a pronounced lack of high-quality, open-access labeled *financial* fake news data, and second, this scarcity necessitates addressing the effects of domain shift, a well-documented phenomenon in the literature (Farahani et al., 2021; Calderon et al., 2024).

Our FinFakeBERT models seek to address this research gap. Given the availability of numerous open cross-domain fake news datasets, we combined several of them into a large dataset (referred to as “Datacorpus”) and fine-tuned the bert-base-uncased model using it. We then compared the resulting model, CDFakeBERT, with a set of baseline models. Here, we observed that CDFakeBERT outperformed all the baseline models we trained and optimized using our data.

Furthermore, we analyzed the effect of domain shift, measured by the false positive rate, which naturally arises in financial fake news detection due to the absence of openly available labeled financial fake news data. We observed that further fine-tuning CDFakeBERT using our carefully curated set of verified fake news and an equal amount of real news significantly reduces the false positive rate, resulting in improved models. Compared to other models in the literature, ours are trained on a broader and more diverse dataset. For instance, FakeBERT (Kaliyar et al., 2021) was trained with less than 10 percent of the data used for CDFakeBERT.

It is crucial to conduct research on the detection of financial fake news, owing to the immense negative impact it has on investors, markets, and society as a whole. Studies examining the impact of fake news on financial markets often rely on very small amounts of non-generalizable data, thereby limiting the benefits for real-world applications.

In addition, we evaluated our model's performance on new, unseen data and found that it is either superior to or only slightly outperformed by models specifically trained on that data. When testing openly accessible models from Hugging Face on the Bloomberg dataset (which contains only real news), we found that our model clearly outperforms them by exhibiting a much lower false positive rate (see Table 8). This is vital, as the majority of online financial news articles are genuine; however, a small proportion of fake financial news can cause lasting damage.

### 4.1 Limitations

Although this research makes a significant contribution to the study of financial fake news, certain critical limitations remain that require further research. Our curated, verified fake news dataset consists of only 233 data points. While this helps to reduce the false positive rate when tested on real news, a larger dataset is very likely to lead to even better generalization.

Furthermore, all verified instances of fake news in our dataset were identified through SEC charges. While these constitute a relevant subset of actual financial fake news, other forms of financial misinformation may not surface through SEC proceedings. As a result, the trained models could develop thematic biases, potentially limiting performance when classifying texts with different content.

Analyzing the false positive rate on real financial news provides a good starting point for applying explainability methods to understand why certain texts are misclassified as fake. Future research could also examine the stability of FinFakeBERT more systematically, accounting for both temporal domain shifts and topical variation.

## Data Availability

The raw data supporting the conclusions of this article will be made available by the authors, without undue reservation, insofar as sharing does not violate third-party copyright or privacy-related restrictions.

## Footnotes

1 Also referred to as false or junk news.

2
https://www.businessinsider.com/stock-market-trump-tariffs-90-day-pause-sp500-rally-2025-4

3
https://www.sec.gov/news/pressreleases

4
https://www.businesswire.com/

5
https://www.prnewswire.com/

6
https://www.globenewswire.com/

7
https://www.accesswire.com/

8
https://www.prlog.org/

9
https://finance.yahoo.com/

10
https://web.archive.org/

11
https://www.bloomberg.com/

12
https://www.fool.com/

13
https://www.ft.com/

14
https://huggingface.co/jy46604790/Fake-News-Bert-Detect

15
https://huggingface.co/mrm8488/bert-tiny-finetuned-fake-news-detection

16
https://huggingface.co/XSY/albert-base-v2-fakenews-discriminator

17
https://www.kaggle.com/datasets/clmentbisaillon/fake-and-real-news-dataset

18
https://huggingface.co/therealcyberlord/fake-news-classification-distilbert
