# Supplementary material for: FinFakeBERT: financial fake news detection
Source: Front Artif Intell. 2025 Dec 3;8:1604272. doi: 10.3389/frai.2025.1604272 (PMC12708890; doi:10.3389/frai.2025.1604272)
Supplement: Supplementary file 1 [file Data_Sheet_1.pdf]

Appendix A. Further experiments

Model	Accuracy	Precision	Recall
CDFakeBERT	**98.3%**	**98.9%**	**97.6%**
SVM	95.7%	95.2%	95.9%
DNN	95.7%	95.6%	95.4%
PA	94.8%	94.4%	94.7%
MNB	88.7%	91.4%	84.2%
RFC	86.9%	90.4%	81.4%

Table A.1: Comparison of baseline models (support vector machine (SVM), deep neural network (DNN), multinomial naive Bayes classifier (MNB), passive aggressive classifier (PA), and random forest classifier (RFC)) with the fine-tuned BERT model, using an 80/20 train–test split.

Appendix B. Summary of Studies on Financial Fake News Detection.

Paper	Model	Dataset fake, size and type	Dataset legit, size and type	Performance	Domain Shift Analysis	Fake news verification method
(Kogan et al., 2023)	LIWC algorithm (Linguistic Inquiry and Word Count model)	171 Fake News (Rick Pearson, SEC identified)	Seeking Alpha and Motley; 334 legit news (same authors as fake)	—	No	Based on trust – the fake news were assumed fake because Pearson and the SEC identified them.
(Clarke et al., 2021)	Gradient boosting	383 Fake News (Seeking Alpha, SEC-related, companies charged)	157,253 legit news (Seeking Alpha)	Mean Precision: 0.871, Recall: 0.905, F1: 0.887	No	SEC lawsuits reviewed and related fake news collected on Seeking Alpha.
(Zhi et al., 2021)	Multi-Fact-CNN-LSTM-Model	8,000 labeled articles (from financial news websites such as East Money Information, Sina, Headline, etc.)	Multi-Fact-CNN-LSTM-Model	Accuracy: 0.921, Precision: 0.889, Recall: 0.956, F1: 0.923	No	Source verification using a list of trusted websites, market data analysis, and user comments.
(Zhang et al., 2022)	TDT-based Model	383 Fake News (from Clarke et al. (2021))	6,866 legit news (from various financial sources like Seeking Alpha, SEC, StockTwits, Twitter, and Compustat)	Precision: 0.900, Recall: 0.994, AUC: 0.895, F1: 0.945	No	Refer to Clarke et al. (2021) for details.
(Chung et al., 2023)	TRNN (Temporal Recurrent Neural Network)	1000 articles (Stocktwits, Twitter)	1000 articles (Stocktwits, Twitter)	**Downward:** Precision: 0.8036, Recall: 0.8223, F-score: 0.8113<br>**Upward:** Precision: 0.7789, Recall: 0.8481, F-score: 0.8108	No	Dataset reviewed by five independent validators.
(Zhang and Liu, 2023)	Deep Learning	160 Fake News (Chinese finance sector)	480 legit news (Wind financial database)	Accuracy: 94.38%, Precision: 86.49%, Recall: 88.89%, F1-score: 87.67%	No	Not specified.

Table (continued)

Paper	Model	Dataset fake, size and type	Dataset legit, size and type	Performance	Domain Shift Analysis	Fake news verification method
(Kamal et al., 2023)	Fin-MisID	3,179 Fake News (Politifact)	1,791 legit news (Politifact)	Precision: 0.82, Recall: 0.88, F-score: 0.85, Train Acc.: 0.88, Test Acc.: 0.83	No	Fake news verified via official corrections on “Cninfo” and subsequent online search.
(Mohankumar et al., 2023)	CAEN and CSRN (cross-linking networks)	5,748 (WELFake) and 1,254 Fake News (Kaggle)	5,743 (WELFake) and 1,254 Fake News (Kaggle)	WELFake: Precision: 0.94, Recall: 0.95, F-score: 0.94, Kaggle: Precision: 0.85, Recall: 0.90, F-score: 0.87	No	Datasets were labeled.
(Rangapur et al., 2025)	FIN-FACT	1,492 false claims	1,275 true claims	–	No	Labelled by Politfact, Snopes, FactCheck

Table B.2: Summary of studies on financial fake news detection.

WELFake is a dataset for fake news detection with 72,134 news articles (37, 106 fake, 35,028 real). It combines content from four sources (Kaggle Fake News, Mclntire Fake News, Reuters, BuzzFeed Political). We selected only news items from the financial sector, resulting in 5,748 fake and 5,743 legitimate financial news items.

Fin-Fact is a specially curated dataset for finance-related fact checks with 3,369 claims from areas such as economy budget, income, taxes, and debt. It combines claim text, author publication date, justifications, evidence and image links, and truth labels (True, False, NEI). The claims come from PolitiFact and Snopes, have been filtered, cleaned up, and supplemented with structured metadata Of the total 3,369 claims, 1,275 are marked as True, 1,492 as False, and 602 as NEI (Not Enough Information). This makes the dataset particularly suitable for analyzing and training fact-checking models in the financial sector.

References

Chung, W., Zhang, Y., Pan, J., 2023. A theory-based deep-learning approach to detecting disinformation in financial social media. Information Systems Frontiers 25, 473–492.

Clarke, J., Chen, H., Du, D., Hu, Y.J., 2021. Fake News, Investor Attention, and Market Reaction. Information Systems Research 32, 35–52. URL: https://pubsonline.informs.org/doi/10.1287/isre.2019.0910, doi:10.1287/isre.2019.0910.

Kamal, A., Mohankumar, P., Singh, V.K., 2023. Financial misinformation detection via roberta and multi-channel networks, in: International Conference on Pattern Recognition and Machine Intelligence, Springer. pp. 646–653.

Kogan, S., Moskowitz, T.J., Niessner, M., 2023. Social media and financial news manipulation. Review of Finance 27, 1229–1268.

Mohankumar, P., Kamal, A., Singh, V.K., Satish, A., 2023. Financial fake news detection via context-aware embedding and sequential representation using cross-joint networks, in: 2023 15th International Conference on COMmunication Systems & NETworkS (COMSNETS), IEEE. pp. 780–784.

Rangapur, A., Wang, H., Jian, L., Shu, K., 2025. Fin-fact: A benchmark dataset for multimodal financial fact-checking and explanation generation, in: Companion Proceedings of the ACM on Web Conference 2025, pp. 785–788.

Zhang, N., Liu, J., 2023. Fake financial news detection with deep learning: Evidence from china. Procedia Computer Science 221, 154–160.

Zhang, X., Du, Q., Zhang, Z., 2022. A theory-driven machine learning system for financial disinformation detection. Production and Operations Management 31, 3160–3179.

Zhi, X., Xue, L., Zhi, W., Li, Z., Zhao, B., Wang, Y., Shen, Z., 2021. Financial fake news detection with multi fact cnn-lstm model, in: 2021 IEEE 4th International Conference on Electronics Technology (ICET), IEEE. pp. 1338–1341.
